# Study on the Drug Targets and Molecular Mechanisms of *Rhizoma Curcumae* in the Treatment of Nasopharyngeal Carcinoma Based on Network Pharmacology

**DOI:** 10.1155/2020/2606402

**Published:** 2020-03-28

**Authors:** Sijia Zhai, Qiao Huang, Xingwei Liao, Shihua Yin

**Affiliations:** Department of Otolaryngology ＆ Head and Neck Surgery, The Second Affiliated Hospital of Guangxi Medical University, Nanning City, Guangxi Zhuang Autonomous Region 530000, China

## Abstract

**Aim:**

To analyse the target of *Rhizoma Curcumae* in nasopharyngeal carcinoma by using network pharmacological techniques and to explore the associated molecular mechanism.

**Methods:**

The targets of nasopharyngeal carcinoma were retrieved from the GeneCards database. At the same time, the drug therapeutic targets of *Rhizoma Curcumae* were obtained from the TCMSP and SymMap databases. The data were imported into the STRING database and Cytoscape 3.7.1 to construct a network of “Chinese medicine component-target-disease” interactions; then, the intersection was screened as the core *Rhizoma Curcumae* antinasopharyngeal cancer targets. Through GO target function and KEGG pathway enrichment analyses of the core targets, we predicted the biological processes and key signalling pathways involved in the *Rhizoma Curcumae* treatment of nasopharyngeal carcinoma.

**Results:**

Twenty-five core targets of *Rhizoma Curcumae* in nasopharyngeal carcinoma were mined: TP53, BCL2 ICAM1 RXRA, TLR3 and TLR9, TNF, PTGS2, IL-6, CTSD, MMP2, MMP9, MMP14, TIMP2, ABCC1, ABCB1, ABCG2, and so on. The results of visual analysis showed that the *Rhizoma Curcumae* treatment of nasopharyngeal carcinoma mainly involves leukocyte adhesion to vascular endothelial cells, positive regulation of NF-*κ*B import into the nucleus, regulation of the reactive oxygen species biosynthetic and metabolic process, regulation of the chemokine biosynthetic and metabolic process, various cancer-related signalling pathways, and a variety of cytokine signal transduction pathways, such as the NF-*κ*B, TLR, IL-17, and TNF signalling pathways.

**Conclusion:**

The core targets predicted by our research can be used as molecular markers for the treatment and prediction of nasopharyngeal carcinoma. The mechanism of *Rhizoma Curcumae* treatment in NPC may be related to immune regulatory pathways, the inhibition of cancer cell proliferation, metastasis, and angiogenesis, as well as the regulation of tumour microenvironment. Combined with the prediction of its associated mechanism of action, the core targets can provide targeted reference value for subsequent drug development related to *Curcuma*.

## 1. Introduction

Nasopharyngeal carcinoma (NPC) is a common epithelial tumour in southern China and Southeast Asia. It is one of the most common malignant tumours in China. The incidence of NPC is closely related to heredity, EB virus infection, and environmental factors. Lymph node metastasis can occur early, with most patients first diagnosed at the late stage; NPC has a high recurrence rate and easy distant metastasis [1], resulting in a reduction in the therapeutic effect and 5-year survival rate of patients with NPC. Conventional conformal radiotherapy (CCRT) plus adjuvant cisplatin-based chemotherapy reached consensus on the mainstay treatment for NPC [2], but combined treatment can produce serious adverse reactions, coupled with chemotherapy drug resistance, resulting in unsatisfactory treatment. However, in combination with traditional Chinese medicine treatment, long-term conditioning and repair for treatment-related side effects can be performed.

Traditional Chinese medicine (TCM) with antitumor effects has become an important part of clinical prescriptions of TCM and the source of new antitumor drug development [3]. *Rhizoma Curcumae*, known as Ezhu in China, is a plant of *Curcuma* L of Zingiberaceae, from dried roots of *Curcuma phaeocaulis*, *Curcuma kwangsiensis*, and *Curcuma wenyujin*. It is a traditional Chinese medicine, widely distributed throughout China, and contains a variety of biologically active ingredients, such as curcumol [4]. It has been reported to have low toxicity against tumours, with antiproliferative, anti-inflammatory, antibacterial, antioxidant, and antifibrotic effects [5]. *Rhizoma Curcumae* has been used in the clinical treatment of cervical cancer and has been proven to have antitumor effects in various human cancers such as liver cancer, ovarian cancer, and breast cancer [6, 7]. *Rhizoma Curcumae* is a common medicine in the traditional Chinese medicine treatment of NPC. Studies have shown that curcumol may inhibit the further development of NPC by attenuating epithelial-mesenchymal transition in nasopharyngeal carcinoma cells [8]. Curcumol can inhibit the proliferation of nasopharyngeal carcinoma cells and induce apoptosis [9]. These studies indicate that *Rhizoma Curcumae* has significant antitumor effects, but its target in NPC cells remains unclear. Revealing and understanding the mechanism of the action of *Rhizoma Curcumae* is essential for the prevention and treatment of nasopharyngeal carcinoma.

Network pharmacology is a new drug design method and strategy based on the rapid development of systems biology and multidirectional pharmacology. It is based on the “disease-gene-target-drug” interaction network. Network pharmacology provides a new approach for studying the mechanism of Chinese medicine ingredients through identifying connections with targets and diseases; through the analysis of information from existing databases, such as gene network, protein network, disease network, and drug network libraries, combined with spectral data obtained from our experiments, we used professional network analysis software and algorithms to apply this approach. At present, there are few studies on the target and molecular mechanism of *Rhizoma Curcumae* treatment in NPC. For further exploration and integration, our study used network pharmacology to construct a network of “component-target-disease-pathway” interactions to predict the targets of *Rhizoma Curcumae* treatment and its possible mechanism of action in NPC, providing a reference for subsequent experiments and even a new direction for molecular targeted therapy of NPC.

## 2. Materials and Methods

### 2.1. Screening of Active Ingredients and Targets

The TCMSP database and the SymMap database were used to obtain the active ingredients and the corresponding drug targets of *Rhizoma Curcumae*, with oral availability (OBioavail, OB) ≥30% and drug-like properties (drug-likeness, DL) ≥0.18 as a standard screen for chemical components acting as active ingredients in *Rhizoma Curcumae*. We used the GeneCards database to find targets related to NPC, screening genes with a correlation score of ≥5 as candidate target genes. We used the UniProt database to remove non-human target gene names, and finally, we eliminated duplicates.

### 2.2. Construction of a *Rhizoma Curcumae*-Target-Nasopharyngeal Carcinoma PPI Network

Disease and target PPI data were obtained from the STRING database, PPI data with a score of ≥0.9 were screened, and the results were imported into Cytoscape 3.7.1 to construct a *Rhizoma Curcumae*-target-nasopharyngeal cancer PPI network.

### 2.3. Acquisition of Antinasopharyngeal Carcinoma Targets of *Rhizoma Curcumae*

The targets of *Rhizoma Curcumae* and the targets of nasopharyngeal carcinoma were mapped to each other to obtain the targets of *Rhizoma Curcumae* treatment for NPC. The antinasopharyngeal cancer targets of *Rhizoma Curcumae* obtained after mapping were imported into the STRING database to determine the role between the target proteins and their functions. PPI data with a score of ≥0.9 were screened and visualized by Cytoscape 3.7.1. Finally, we determined the core target of *Rhizoma Curcumae* treatment in NPC.

### 2.4. Construction of the Component-Target-Disease-Pathway Interaction Network

Pathway enrichment analysis of the core target genes of *Rhizoma Curcumae* against NPC was performed. The target-pathway network and component-target network as well as *Rhizoma Curcumae*-target-NPC were imported into Cytoscape 3.7.1 for network consolidation. The integration result of the “component-target-disease-path” was obtained.

### 2.5. GO and KEGG Analyses of Core Targets

The Cytoscape 3.7.1 plug-in ClueGO was used to perform GO and KEGG analyses on the core targets of Rhizoma Curcumae against nasopharyngeal carcinom and to visualize the possible biological processes, molecular function, cellular component, and signalling pathways that were involved.

## 3. Results

### 3.1. Target Information Acquisition

We collected 681 genes as the study targets by using the GeneCards database. Through TCMSP, we screened the active ingredients of *Rhizoma Curcumae* with the DB ≥ 30 and DL ≤ 0.18 standards and found 33 corresponding active ingredients. 40 active ingredient targets were obtained by using the SymMap database. Subsequently, the UniProt database was used for target standardization and removal of nonhuman targets and duplicates. Ultimately, we obtained 66 *Rhizoma Curcumae*-related targets.

### 3.2. PPI Network Construction of *Rhizoma Curcumae*-Target-NPC

**Figure 1:**
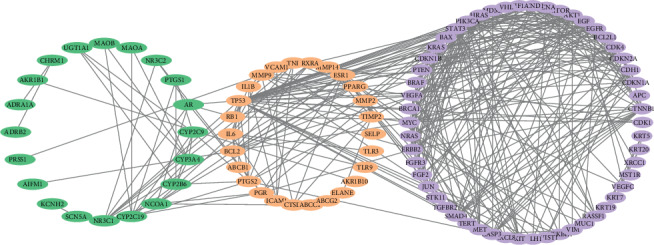
Target-disease network (green is the target of the Rhizoma Curcumae drug, purple is the target of NPC, and orange is the antinasopharyngeal cancer target of *Rhizoma Curcumae*).

The PPI data of the disease targets and the active targets of *Rhizoma Curcumae* were obtained through the STRING database, and the PPI data with confidence scores ≥0.9 were selected and imported into Cytoscape to construct the target network interaction map. The merge function was used to merge the target network map with the disease target network map to obtain a target-disease network map consisting of 100 nodes and 359 edges (Figure 1).

### 3.3. Interaction Network of Antinasopharyngeal Carcinoma Targets of *Rhizoma Curcumae* and Function-Related Proteins

**Figure 2:**
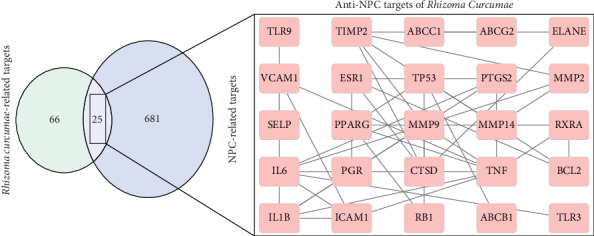
Interaction network of curcuma zedoary antinasopharyngeal carcinoma targets and function-related proteins.

The targets obtained by mapping the targets of *Rhizoma Curcumae* and the targets of NPC to each other were converted into PPI data. Then, the PPI data with confidence scores ≥0.9 were imported into the Cytoscape to construct a network of the antinasopharyngeal cancer targets of *Rhizoma Curcumae* and function-related protein interactions. Finally, 25 core targets were obtained, namely, SELP, TLR3, TLR9, ELANE, ABCG2, ABCC1, CTSD, ICAM1, PGR, ESR1, PTGS2, ABCB1, IL-6, BCL2, RB1, TP53, IL1B, MMP9, VCAM1, TNF, PPARG, RXRA, MMP14, MMP2, and TIMP2. As shown in Figure 2, the network map had 25 nodes, which had 41 edges indicating interactions between the nodes.

### 3.4. Construction of the Component-Target-Disease-Pathway Interaction Network

**Figure 3:**
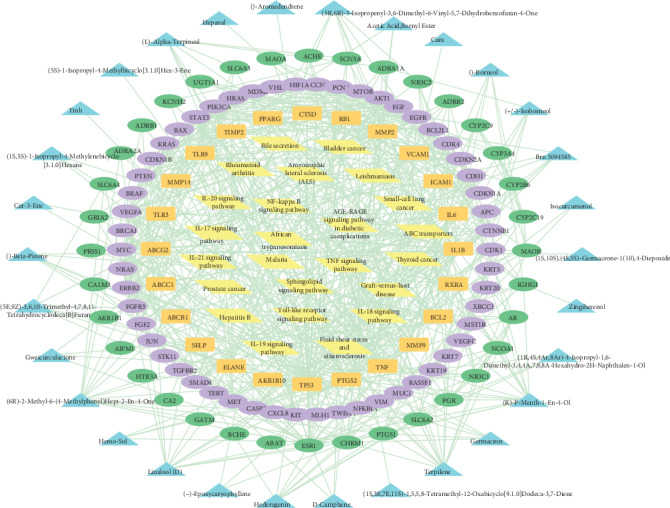
Component-target-disease-pathway interaction network (blue 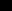 is the drug active ingredient, green 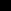 is the target of *Rhizoma Curcumae*, purple 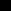 is the target of NPC, orange 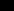 is the antinasopharyngeal cancer target of *Rhizoma Curcumae*, and yellow 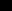 is the enriched signal pathway).

Using the merge function in Cytoscape, we obtained the component-target-disease-pathway interaction network. As shown in Figure 3, the drug active ingredient at the outermost circle corresponds to the drug target at the inner circle which interacts with the disease target to obtain the core target. Finally, in the center of the circle, the enriched signal pathways were predicted by the core target. There are 168 nodes and 606 edges in the network.

### 3.5. GO and KEGG Analysis of Core Targets

The Cytoscape plug-in ClueGO was used to visually analyse the function of 25 predicted targets of *Rhizoma Curcumae* in NPC. The results of GO biological function and KEGG signal enrichment analyses were screened according to the *P* value.

**Figure 4:**
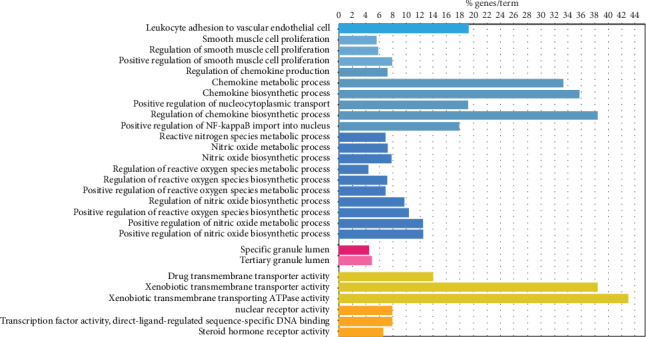
Results of biofunctional enrichment analysis.

The results of GO analysis including biological process, cellular component, and molecular function analysis are shown in Figure 3. The top 20 biological processes of the core targets were mainly involved with leukocyte adhesion to vascular endothelial cells, positive regulation of NF-*κ*B import into the nucleus, positive regulation of nucleocytoplasmic transport, regulation of the reactive nitrogen species metabolic process, regulation of the reactive oxygen species biosynthetic and metabolic process, proliferation of smooth muscle cells, regulation of the chemokine biosynthetic and metabolic process, and regulation of the nitric oxide biosynthetic and metabolic process, suggesting that curcuma may play a role against NPC by regulating the above biological processes (Figure 4, blue parts). As for molecular function, the core targets were enriched in drug transmembrane transporter activity, xenobiotic transmembrane transporter activity, nuclear receptor activity, transcription factor activity, direct ligand-regulated sequence-specific DNA-binding, steroid hormone receptor activity (Figure 4, yellow parts). In addition, with regard to cell components, the core targets were enriched in the tertiary granule lumen and specific granule lumen (Figure 4, red parts).

**Figure 5:**
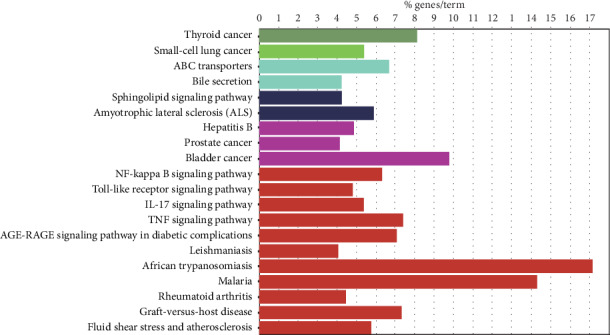
Enrichment analysis results of signalling pathways.

The results of the top 20 signal pathway enrichment analysis are shown in Figure 5. The results indicated that the KEGG pathways of the top targets are mainly involved in the thyroid cancer, small-cell lung cancer, ABC transporter, bile secretion, sphingolipid signalling, amyotrophic lateral sclerosis (ALS), hepatitis B, prostate cancer, bladder cancer, NF-*κ*B signalling, toll-like receptor signalling, IL-17 signalling, TNF signalling, AGE-RAGE signalling in diabetic complications, leishmaniasis, African trypanosomiasis, malaria, rheumatoid arthritis, graft-versus-host disease, and fluid shear stress and atherosclerotic pathways. These pathways are closely related to the regulation of various cancer and cytokine signal transduction pathways, suggesting that curcuma may have an anticancer effect by regulating the above pathways.

## 4. Discussion

To find the key target of curcuma zedoary in the treatment of nasopharyngeal carcinoma, this research explored the mechanism of action of *Rhizoma Curcumae* against NPC by means of network pharmacology and found its multitarget and multifunctional characteristics. A total of 66 curcuma zedoary targets and 681 nasopharyngeal carcinoma targets were mined. After mapping, 25 curcuma zedoary treatment targets for nasopharyngeal carcinoma were obtained: TP53, BCL2, ICAM1 RXRA, TLR3 and TLR9, TNF, PTGS2, IL-6, CTSD, MMP2, MMP9, MMP14, TIMP2, ABCC1, ABCB1, ABCG2, and so on.

TP53 is a broad-spectrum tumor suppressor gene involved in cell growth and apoptosis, and experiments have confirmed that TP53 accumulation is found in NPC tissue, the function of which may be out of control [10]. NF-*κ*B pathway is one of its downstream regulatory pathways [11], which is a key node for amplifying inflammatory signals in EBV-infected epithelial cells. Both BCL2 and ICAM1 are downstream target genes of the NF-*κ*B signalling pathway that are associated with proliferation and metastasis of NPC [12, 13]. BCL2, as a target gene for growth and development of nasopharyngeal carcinoma cells [14], is a poor prognostic marker for nasopharyngeal carcinoma [15]. Studies have found that RXRA is upregulated in one type of NPC and may be associated with an immune response [16]. EBV-infected NPC cells can activate the TLR3 and TLR9/NF-*κ*B signalling pathways to promote immune cells to secrete a large number of cytokines, chemokines, and growth factors such as TNF*α* [17, 18], forming a complex tumour microenvironment which is associated with the development of nasopharyngeal carcinoma [19], so the upregulation of TNF is often associated with NPC metastasis and low survival rates. PTGS2 is also involved in the NF-*κ*B pathway and mediates the expression of proangiogenic cytokines which promotes angiogenesis in NPC [20]. Elevated levels of IL-6 have been verified by Ke et al. [21, 22] to be associated with NPC metastasis and low survival rates. IL-6 is a well-known proangiogenic factor that promotes tumour cell proliferation, angiogenesis, and invasion [23]. Recent studies have shown that the concentration of CTSD in the serum of NPC patients is significantly increased and has diagnostic value [24]. Combined with gene chip technology, CTSD was found to be involved in the cell proliferation, apoptosis, invasion, and metastasis of NPC [25].

It was found that MMP is highly expressed in NPC tissue, and the high expression of MMP indicates poor prognosis of NPC patients. The three subtypes of matrix metalloproteinases (MMPs), MMP2, MMP9, and MMP14, can promote the invasion and metastasis of NPC cells [26, 27]. MMP2 is mediated by NF-*κ*B [28], and MMP2/9 is involved in the ERK1/2-MMP2/9 extracellular signal-regulated kinase signalling pathway [29]. Tissue inhibitor of metalloproteinase-2 (TIMP2) is an upstream gene of MMP2 [30], and the imbalance of MMP2/TIMP2 may have prognostic value for nasopharyngeal carcinoma. The results of immunohistochemistry analysis showed that the proportion of cells stained positive for TIMP2 in NPC was significantly higher than that in inflammatory tissues, and the proportion of positive cells increased significantly with lymph node metastasis. TIMP2 may play a key role in facilitating the degradation of the basement membrane and the invasion of surrounding tissues in cancer cells to form metastatic colonies in lymph nodes [31]. ABCB1, ABCC1, and ABCG2 belong to the ATP transporter gene family, which is related to NPC drug resistance. The expression of ABCC1 and ABCG2 in NPC tissues is significantly higher than that in normal nasopharyngeal tissues by real-time PCR [32]. ABCB1 gene polymorphism and ABCG2 upregulation may be responsible for the resistance of NPC cancer stem cells to chemotherapeutic drugs [33, 34]; tumour suppressor gene IRF6 kills cancer stem cells in NPC by targeting the ABCG2 gene [35].

In addition, we performed GO annotation analysis and KEGG pathway analysis by target proteins. GO analysis showed that the biological processes of core targets are mainly focused on leukocyte adhesion to vascular endothelial cells. Cell-cell adhesion is the basis of tissue integrity [36]. Decreased cell adhesion and enhanced motor function facilitate tumor cells to escape from the primary site and metastasize [37]. *Rhizoma Curcumae* can be used as an antinasopharyngeal cancer agent by positively regulating the metabolic process of reactive oxygen species biosynthesis. TP53, one of the core targets screened out, can also enhance the damage of reactive oxygen species (ROS). So, *Rhizoma Curcumae* maybe reduce reactive oxygen species damage by regulating TP53 to play role against nasopharyngeal carcinoma, the mechanism of which needs further research. Besides, *Rhizoma Curcumae* is likely to inhibit tumour microenvironment formation by regulating the biochemical metabolic processes of chemokines.

The KEGG analyses showed that most of the enriched biological information was associated with the thyroid cancer pathway, small-cell lung cancer pathway, prostate cancer pathway, bladder cancer pathway, and other cancer pathways. According to an analysis of the literature, we found that multiple target proteins contained in these pathways can activate the MAPK signalling pathway [38–41]. Therefore, we speculated that *Rhizoma Curcumae* may regulate the proliferation of nasopharyngeal carcinoma cells by regulating the MAPK signalling pathway. NF-*κ*B, as the key node, and its upstream TP53 gene as well as downstream BLC2 and ICAM1 were found closely related to NPC. EBV infection can activate TLR3 and TLR9/NF-*κ*B signalling pathways to upregulate TNF. And the abnormally regulated toll-like receptor signalling pathway, TNF signalling pathway, and NF-*κ*B signalling pathway as well as positive regulation of NF-*κ*B import into the nucleus are closely related to the occurrence and development of NPC. These signal pathways and targets will guide future research.

Moreover, the results of KEGG analysis were also enriched in the IL-17 signalling pathway, hepatitis B pathway, AGE-RAGE signalling in the diabetic complication pathway, and malaria pathway. IL-17 is a proinflammatory cytokine mainly produced by T-helper 17 cells. It is reported that IL-17A promotes the migration and invasion of NPC cells through the p38 mitogen-activated protein kinase/NF-*κ*B signalling pathway, which subsequently upregulates the expression of MMP2 and MMP9 and enhances epithelial-mesenchymal transition [42]. Hepatitis B virus infection is an independent risk factor for early NPC, which may be related to decreased immune function. It is reported that the prognosis of HBsAg (+) NPC patients is poor, and distant metastasis, especially liver metastasis, is more common in these patients [43]. Anti-HBV treatment may improve the prognosis of HBV-infected NPC patients [44]. Type 2 diabetes, a risk factor for NPC, is associated with a poor prognosis in NPC [45]. High-titer malaria antibodies can diagnose NPC.

## 5. Conclusion

In summary, we can speculate that *Rhizoma Curcumae* exerts potent anticancer effects by regulating the expression of the predicted targets in nasopharyngeal carcinoma cells. These predicted targets can also be used as potential molecular markers against nasopharyngeal carcinoma. The pharmacological mechanism of *Rhizoma Curcumae* in NPC may be closely related to the regulation of tumor proliferation and metastasis as well as tumour environment, cancer pathways, and transduction of some cytokine signalling pathway, indicating that the pharmacological and molecular mechanisms generated via the network pharmacology approach are predictive. The accuracy and practicality of this approach lay a good foundation for further exploration of the pharmacological targets and molecular mechanisms of *Rhizoma Curcumae* in nasopharyngeal carcinoma.

## Data Availability

The data used to support the findings of this study are available from the corresponding author upon request.
